# The Shelter Problem

**Published:** 1940

**Authors:** 


### THE SHELTER PROBLEM.

Early in September, 1940, the Government's attention was drawn to the danger to the public health caused by the conditions of many of the large public shelters. A small Committee to inquire into this state of affairs, and to make recommendations, was set up jointly by the Ministry of Home Security and the Ministry of Health on 14th September, with Lord Horder as Chairman.

The crux of the position in its physical aspects was gross overcrowding. For this the remedy was, of course, dispersal, and the various ways in which this could be effected were dealt with in the recommendations of the Committee—by popularizing the domestic and communal shelters, by improving their amenities, by restoring the popularity of the Anderson shelters, by reconditioning them where this had become necessary, by combing the Boroughs for alternative large basement shelters of a type the public affect and, if needs be, by requisitioning factory basements, and by urging evacuation of children, the aged and the infirm to the limits of the voluntary basis. But inasmuch as dispersal involved a long-term policy, and it was imperative to combat the hygienic hazard at once, a short-term policy of conditioning the large public shelters in respect of ventilation, sanitation, bunking, the provision of medical-aid posts, daily medical inspection and the appointment of paid shelter wardens, was also urged. Unfortunately, it had been no one's business to bring these places under control, to inspect them, or to fit them for the purposes to which the public was putting them.

In the ordering of this difficult problem Lord Horder's Committee has been able to exercise a considerable personal influence, collectively and individually, and has also been able to keep up a sustained attack upon the more serious dangers and delays. By unremitting personal attention, by frequent visits to shelters, by interviews with Ministers and Regional Commissioners, and by actual contact with officials, it has proved possible to hasten considerably the implementation of the Committee's recommendations.

The " hard core " of the shelter population presents a problem of a wider sort than the mere threat to the common health through gross insanitary conditions. This is the problem of the slow emergence of the slum habitué from the casual shelterer, and the tendency of a merely opportune device for affording protection from air attack to develop into an underground residence. Clearly this matter overlaps very definitely with public health interests.

New abuses arise, hydra-headed, in these large public shelters almost whilst one is looking, and they must be dealt with promptly and firmly in the interest of the common health. Control is the key to the situation.

Obviously every public shelter should be either recognized and scheduled or disallowed. The worst conditioned shelters to-day are neither coded nor closed down. They are a sort of no man's land, and this abuse tends to increase, so that mere protection is not, in some of them, their chief *raison d'être*.

The shelter situation presents essentially a task for administrative medicine. By administrative medicine is meant, primarily, the work of the Medical Officers of Health and their staffs. More and more does Lord Horder's Committee look to these men and women for the implementing of its recommendations. Not directly, because they are the servants of their Local Authorities, but their indirect influence cannot be over-estimated. However, in dealing with the shelterers themselves, and in stimulating these people to an attitude of self-help, the doctor's influence is second to none. Shelter life to-day is providing a unique field for health education and it behoves us to explore it to the full.

Dr. R. H. PARRY, Medical Officer of Health for Bristol, has very kindly furnished the following notes:—

The greatest difficulty in the shelter "problem" is the adjustment of the balance between considerations of health and physical safety from bombs; in many ways these two are incompatible. There are these main groups into which most types of shelters, other than domestic, may be placed.

1. Standard surface shelters built by Local Authority according to Ministry of Home Security specification.

2. Cellars, basements, church crypts, factory shelters.

3. "Natural" shelters—tunnels, caves, disused workings, railway arches.

It is the duty of the Local Authority to schedule all public shelters, but before scheduling any shelter the Local Authority must be satisfied that the required standard of protection and hygienic safeguard are secured. Difficulties arise with the second and third groups, and especially in the third group, which shelters in many cases do not give the standard protection and cannot be sufficiently adapted to give reasonably hygienic safeguards. As far as the Local Authority is concerned, it has full control over all shelters which it has built itself under the Ministry of Home Security instruction, and over those shelters which it has requisitioned or taken over from firms, factories and other sources. There are certain private firms which own shelters in factories which have offered accommodation to the general public during certain hours. The Local Authority has no power to interfere with the conduct of these shelters unless it requisitions them, and in this connection it must be noted that certain firms, including Government departments, cannot be dealt with in this way by the requisitioning powers of the Council. However, as a general rule, such shelters are good ones, and it is in the firms' own interests that discipline in them is good. The real trouble arises with the so-called "natural" shelters—Group 3. In many cases the public have great faith in these shelters, even though this faith is not supported by expert engineering opinion as to safety. Such shelters do not lend themselves to alteration for hygienic or sanitary purposes, and the results are often deplorable. Indeed, some of these shelters, for example disused workings, may be so impossible of adaptation, that nothing can be done.

Within the limits here set out, the Local Authority has great powers. It can regulate the number of people to be admitted to a shelter and the number sleeping in a shelter, and it can regulate the admission of people suffering from infectious disease or who are verminous or offensively unclean. Bunks are provided for sleeping, and the Council allocate tickets for each person occupying a bunk.

The Council will not schedule any shelter as a public shelter unless it conforms to the standard of protection laid down, and hygienic safeguard secured. The Medical Officer of Health has the power to inspect those shelters scheduled by the Council. With those not so requisitioned, power to inspect and control would only be possible should it be necessary for the prevention of disease.

With regard to the provision of medical aid, twelve medical practitioners have been appointed—two for each division or district, *i.e.* Clifton, St. George, Central, Shirehampton, Knowle, Bedminster. These doctors will be on call for the shelters in their district for emergency purposes mainly (including confinements) and must be summoned by a responsible officer, *i.e.* Police Officer, Warden, Shelter Marshal, and not by individual members of the general public. In addition, they may be required by the Medical Officer of Health, from time to time, to visit the shelters for routine health inspection purposes. Forms have been provided, obtainable from Shelter Marshals, which must be filled in by the visiting doctors, giving details of patients seen and the action taken. These report forms are then sent to 40 Prince Street for record purposes and to enable the Medical Officer for the Shelter Management Committee to keep in touch with outbreaks of infectious disease and illness generally in the shelters.

About twenty Medical Rooms or Medical Aid Posts are being constructed in various shelters ; auxiliary nurses will be appointed to co-operate with the doctor and carry out any *minor* treatment necessary to the occupants of the shelter. It must be clearly understood that these Medical Aid Posts in shelters are *not* intended to be used for treating air-raid casualties or street accidents, but solely for the use of the occupants, *i.e.* examination of patients, temporary isolation in some cases and first-aid treatment only for illness or minor injury occurring in the shelter. The doctor attending a patient requiring admission to hospital (including maternity cases) will telephone Central Control for an ambulance.

Recently, the Regional Commissioner has made rules giving powers to the shelter doctors to :—

1. To require a person suspected of infectious disease to submit himself to medical examination.
2. To require a person found by the medical officer to be suffering from infectious disease to be isolated temporarily in the shelter.
3. To require the removal of such person to hospital or to his home or some other suitable place, as the medical officer may consider necessary.

